# Hsp90 Picks PIKKs via R2TP and Tel2

**DOI:** 10.1016/j.str.2014.05.012

**Published:** 2014-06-10

**Authors:** Cara K. Vaughan

**Affiliations:** 1 Institute of Structural and Molecular Biology, Birkbeck College and University College London, Malet Street, London WC1E 7HX, UK

## Abstract

Phosphatidylinositol-3 kinase-like kinases (PIKKs) are dependent on Hsp90 for their activation via the R2TP complex and Tel2. In this issue of *Structure*, Pal and colleagues present the molecular mechanism by which PIKKs are recruited to Hsp90.

## Main Text

The diverse cellular roles of the essential molecular chaperone Hsp90 include cell signaling, protein degradation, genome maintenance, and assembly of transcriptional and translational apparatuses (Li and Buchner, 2013). Specificity for this astonishing breadth in function comes from cochaperones that regulate the ATPase activity of Hsp90 and recruit proteins that require Hsp90 to fold. Together, these small molecular machines allow activation of “client” proteins.

Recent work has revealed that the complexity of cochaperones can be considerably greater than previously realized and that cochaperones themselves can be multi-protein entities. This is exemplified by the R2TP complex that is the focus of work by Pal et al. (2014) in this issue of *Structure*.

The R2TP complex has emerged as a key regulator of cell growth and proliferation. It has characterized functions in (1) the assembly of small nucleolar ribonucleoproteins (snoRNPs), complexes that are required for biogenesis of ribosomal, small nuclear, and transfer RNA in eukaryotes; (2) the cytoplasmic assembly of RNA polymerase II; and (3) the regulation of phosphatidylinositol-3 kinase-like kinases (PIKKs) (see Kakihara and Houry, 2012 and Boulon et al., 2012 for reviews). Pal et al. (2014) focus on the role of the R2TP complex in the maturation of PIKKs (Horejsí et al., 2010; Takai et al., 2010), large atypical serine-threonine kinases that are involved in stress responses. The PIKK family comprises ATM, ATR, DNA-PKcs, and all proteins involved in DNA repair; TRRAP, a protein involved in transcriptional regulation; SMG1, a protein required for nonsense mediated decay; and mTOR, a regulator of cap-dependent translation through phosphorylation of ribosomal effectors (Izumi et al., 2012).

**Figure 1:**
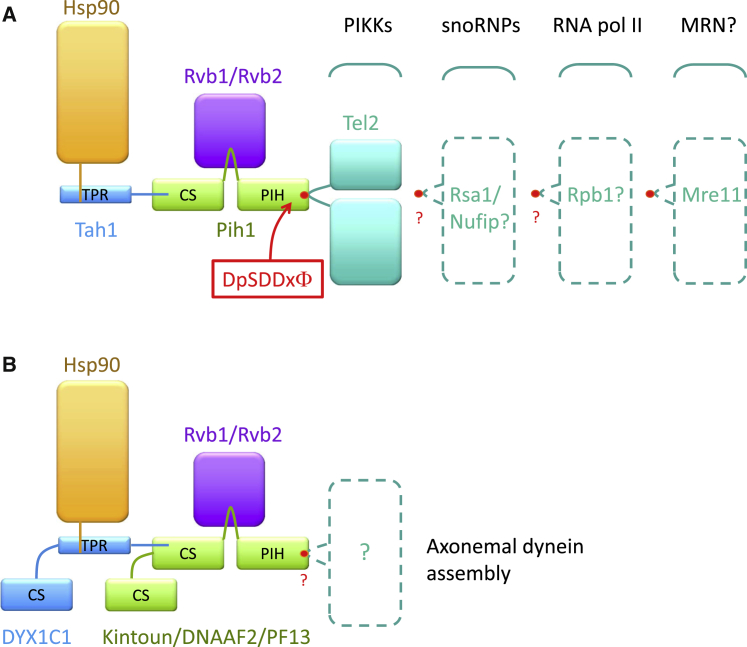
Known and Putative R2TP and R2TP-like Complexes (A) The R2TP complex recruits PIKKs, snoRNPs, and RNA polymerase II to Hsp90. An adaptor protein, Tel2, is required for PIKK recruitment to Pih1 via a phosphoserine motif. Rsa1/Nufip has been proposed to play a similar role for snoRNPs; however, it is unknown whether RNA polymerase II requires a similar adaptor or whether subunits of the complex itself, such Rbp1, interact directly with Pih1. It is also unknown whether the same, or a similar, phosphoserine recognition motif is involved in these interactions (red circle). However, the phosphoserine motif is found in Mre11, which can associate with Pih1; thus, the MRN complex is a putative Hsp90-R2TP client. (B) The complex between DYX1C1 and Kintoun/DNAAF2/PF13 may form an R2TP-like complex linking assembly of axonemal dynein to Hsp90. The protein that associates directly with R2TP to facilitate this interaction is unknown, and it is unknown whether a phosphoserine motif will mediate this.

The core R2TP complex comprises the hexameric AAA+ ATPases Rvb1 and Rvb2, Tah1/Spagh/RPAP3, and Pih1/PIH1D1 (Kakihara and Houry, 2012). All six PIKKs require an additional protein, Tel2, for their association with the R2TP complex (Takai et al., 2010). This is mediated through a direct interaction with Pih1/PIH1D1 (Figure 1A). Through dissection of this complex into interacting units, Pal et al. (2014) present a jigsaw of seven crystal structures that, along with biophysical, biochemical, and genetic data, allow an atomic resolution picture of the R2TP complex and its association with Tel2 to be pieced together. In doing so, a new phospho-serine interaction domain and consensus binding motif are unveiled.

TPR and CS (CHORD and Sgt1) domains, two extremely common folds found among Hsp90 cochaperones, are functionally important for the Tah1-Pih1 complex. Tah1 comprises an N-terminal TPR domain that interacts with the C-terminal MEEVD motif of Hsp90. Its C terminus is disordered in isolation but becomes structured on association with the C-terminal domain of Pih1, which has a CS fold. The metazoan Tah1 ortholog, Spagh/RPAP3, has two tandem TPR domains whose structures are homologous to the Tah1 domain. Biochemical and biophysical analyses suggest that, in yeast, one Tah1 protein associates with each MEEVD motif of the Hsp90 dimer, forming a functional complex with a stoichiometry of (Hsp90)_2_-(Tah1-Pih1)_2_. In mammals, however, the TPR domain duplication results in a stoichiometry of (Hsp90)_2_-(Spagh/RPAP3-PIH1D)_1_, with each of the Spagh tandem TPR domains associating with one of MEEVD motif within the Hsp90 dimer.

Posttranslational modification of Hsp90 and its cochaperones provides an additional layer of regulation of chaperone activity. In particular, phosphorylation of cochaperones by CK2 is common. For instance, CK2 phosphorylation of Tel2 is required for PIKK association with Pih1/PIH1D1 (Horejsí et al., 2010). Pal et al. (2014) present both the apo and phospho-peptide bound forms of the N-terminal Pih1/PIH1D1 domain responsible for the interaction with Tel2. This domain has a novel fold, christened a PIH domain, with a highly charged groove in which the Tel2 phospho-peptide is ensconced. This interaction is unlike known phosphoserine-binding domains; the highly acidic peptide binds with a 3_10_ helical turn and is stabilized by interactions from the three acidic residues that flank the central phosphoserine.

Pal et al. (2014) use the putative consensus motif revealed by the Pih1/PIH1D1-phospho-Tel2 crystal structure in a bioinformatics analysis to search for novel Pih1/PIH1D1 binding proteins. Surprisingly, none of the known proteins involved in the biogenesis of snoRNPs and RNA polymerases were identified in this search. Thus, the question remains as to whether a protein or complex equivalent to Tel2 for PIKKs is required for the association of snoRNPs and RNA polymerases with the R2TP complex, and, if so, whether it binds to Pih1/PIH1D1 via the putative consensus motif (Figure 1A). Nonetheless, one hit obtained has already been validated. A highly conserved sequence motif within Mre11, a component of the MRN (Mre11-Rad50-Nbs1) complex involved in DNA double strand break detection and DNA damage signaling, fulfills the Pih1 consensus-binding motif. MRN function has previously been shown to be Hsp90-dependent; however, the link to R2TP via Mre11 was not previously identified, and therefore this finding suggests an exciting avenue for further research (Figure 1A).

The series of domain interactions revealed in these R2TP structures tantalizingly suggest that other similar cochaperones exist. Kintoun/DNAAF2/PF13, a protein associated with cytoplasmic dynein assembly (Omran et al., 2008) has a PIH and CS domain juxtaposed in a manner similar to that found in Pih1. Kintoun/DNAAF2/PF13 has recently been shown to interact with DYX1C1 (Tarkar et al., 2013), a protein also implicated in dynein assembly and containing a putative Hsp90-interacting TPR domain. Although tentative, the structural information presented by Pal et al. (2014) supports the idea that the Kintoun-DYX1C1 complex may form an R2TP-like Hsp90 cochaperone for the assembly of dynein (Figure 1B).

In conclusion, R2TP and its pattern of TPR, CS, and PIH domain interactions may be a prototype for additional hitherto undiscovered multiprotein Hsp90 cochaperones.
